# Gut ecological networks reveal associations between bacteria, exercise, and clinical profile in non-alcoholic fatty liver disease patients

**DOI:** 10.1128/msystems.00224-23

**Published:** 2023-08-22

**Authors:** Susanne Csader, Xiuqiang Chen, Howell Leung, Ville Männistö, Heikki Pentikäinen, Milla-Maria Tauriainen, Kai Savonen, Hani El-Nezami, Ursula Schwab, Gianni Panagiotou

**Affiliations:** 1 Department of Public Health and Clinical Nutrition, University of Eastern Finland, Kuopio, Finland; 2 Microbiome Dynamics, Leibniz Institute for Natural Product Research and Infection Biology - Hans Knöll Institute, Jena, Germany; 3 Departments of Medicine, University of Eastern Finland and Kuopio University Hospital, Kuopio, Finland; 4 Kuopio Research Institute of Exercise Medicine, Kuopio, Finland; 5 Department of Clinical Physiology and Nuclear Medicine, Kuopio University Hospital, Kuopio, Finland; 6 University of Hong Kong School of Biological Sciences, The University of Hong Kong, Hong Kong, China; 7 Department of Medicine, Endocrinology and Clinical Nutrition, Kuopio University Hospital, Kuopio, Finland; 8 Faculty of Biological Sciences, Friedrich Schiller University, Jena, Germany; 9 Department of Medicine, The University of Hong Kong, Hong Kong SAR, China; North Carolina Agricultural and Technical State University, Greensboro, North Carolina, USA

**Keywords:** gut microbiome, interactome, high-intensity interval training, NAFLD, exercise responsiveness

## Abstract

Gut microbial dysbiosis has been observed in non-alcoholic fatty liver disease (NAFLD). The beneficial impact of exercise, the recommended lifestyle change for NAFLD patients, might be mediated by the gut microbiome (GM). However, the exact taxonomic and functional signatures associated with the host’s clinical and biochemical improvement during exercise in NAFLD patients have not been elucidated yet. To investigate the impact of exercise on GM and reveal GM structures associated with NAFLD improvement during exercise. Stool samples from a 12-week randomized controlled exercise study on NAFLD subjects (*N* = 39) were retrieved, and shotgun metagenomics was performed at baseline and endpoint. Differential correlation network and enrichment analysis were applied to characterize the GM taxonomic and functional changes during the exercise intervention and to associate GM changes with biomarkers of liver status and metabolic dysregulation. Network analysis demonstrated that exercise induced significant changes in the bacterial interactome, which were associated with waist circumference; resting metabolic rate; plasma fasting concentrations of triglyceride, glucose, insulin, and glycated hemoglobin A1c; and homeostasis model assessment for insulin resistance. Grouping the patients in the exercise group (*N* = 20) as responders (*N* = 13) and non-responders (*N* = 7) using their intrahepatic lipid content (IHL) change allowed us to identify bacteria consortia contributing to the levels of alanine fermentation, methanol-, creatinine-, and protocatechuate degradation and as a result to the plasma concentrations of liver injury markers alanine transaminase, gamma-glutamyl transaminase, and aspartate transaminase. We showed that even though exercise has not a significant impact on the alpha- and beta-diversity of NAFLD patients, it leads to a significant re-structuring of the gut bacteria interactome and that specific structural changes can be linked with improvements in IHL.

**IMPORTANCE:**

Our study is applying a community-based approach to examine the influence of exercise on gut microbiota (GM) and discover GM structures linked with NAFLD improvements during exercise. The majority of microbiome research has focused on finding specific species that may contribute to the development of human diseases. However, we believe that complex diseases, such as NAFLD, would be more efficiently treated using consortia of species, given that bacterial functionality is based not only on its own genetic information but also on the interaction with other microorganisms. Our results revealed that exercise significantly changes the GM interaction and that structural alterations can be linked with improvements in intrahepatic lipid content and metabolic functions. We believe that the identification of these characteristics in the GM enhances the development of exercise treatment for NAFLD and will attract general interest in this field.

## INTRODUCTION

Non-alcoholic fatty liver (NAFLD) is the leading cause of chronic liver diseases and is strongly associated with metabolic syndrome (1). NAFLD, which ranges from benign steatosis up to non-alcoholic steatohepatitis (NASH) and can lead to cirrhosis and hepatocellular carcinoma, is significantly associated with increased overall mortality (2, 3). Among multiple pathways and different interactions between the liver and organs, the gut and its microbiome have received increased attention in NAFLD pathogenesis. Small intestine bacterial overgrowth and altered microbiome signatures have been found in NAFLD patients compared to healthy controls (4 – 7). Due to dysbiosis and increased intestinal permeability, gut microbiome (GM)–derived metabolites can reach the liver via the portal vein and influence inflammation and NAFLD progression (1, 8). Furthermore, microbial interactions have several crucial functions for community stability in a healthy microbiome and changes in this interactome can contribute to the host disease (9).

Exercise, in addition to diet, is the recommended treatment for NAFLD has recently been shown to be interconnected with gut microbiota (10 – 12). For example, professional rugby players have higher alpha diversity compared to healthy controls (13) and a half marathon in amateur runners yielded abundance changes in seven taxa and 20 bacterial clades (14). In addition, we previously found differences in gut microbiota associated with insulin sensitivity in prediabetic subjects responding differently to exercise (15). However, strenuous exercise can also induce loss of epithelial integrity and barrier function and splanchnic hypoperfusion with impaired nutrient absorption (16, 17).

While many microbiome studies focused on associations between diseases, low diversity, altered abundances, and composition of specific microorganisms, there is growing interest in the microbial interactome and identifying its keystone taxa in human health and disease (18 – 22). The microbial communities interact as a complex network in which species can cooperate or compete, for instance, for nutrients and metabolites (23). Recent co-occurrence network analysis studies in Crohn’s disease, irritable bowel disease, and obesity showed microbial disturbances and alteration in the GM interactome suggesting an essential role of microbial interactions in disease progression (19, 20). Earlier studies on the GM and NAFLD were predominantly centered on identifying individual gut species and their functions (24 – 27). While those studies have helped appreciate the involvement of gut species in NAFLD development, the complexity of the disease requires therapeutic interventions that move beyond individual species to small consortia.

We recently performed a randomized controlled exercise intervention in participants with NAFLD (28), and well-documented clinical measurements were taken to assess how the phenotypes changed to the exercise intervention. Here, we took a step further to characterize taxonomically and functionally the GM using a comprehensive shotgun metagenomics analysis. In addition, we used here a community approach, through co-abundance ecological networks, to investigate the impact of exercise on GM and reveal GM structures associated with NAFLD improvement during exercise.

## MATERIALS AND METHODS

### Study

The study design has been reported in detail previously (28). In brief, participants with NAFLD diagnosis were randomly assigned to an intervention or control group according to body mass index (BMI), age, gender, and status of glucose metabolism. The inclusion criteria were age 18–70 years and BMI below 35 kg/m^2^. NAFLD diagnosis was made with ultrasound, magnetic resonance imaging (MRI), or computed tomography. The intervention group followed an individualized 12-week high-intensity interval training (HIIT) based on an ergospirometry test. The control group maintained their sedentary lifestyle with no changes in physical activity. The subjects were instructed to keep their dietary habits unchanged. Four-day food records were collected just before the intervention started and at week 11 checked by a clinical nutritionist at return. Intrahepatic lipid (IHL) content was measured by nuclear MRI.

Blood samples were drawn after an overnight fasting. Plasma glucose concentration and lipid profile were analyzed at Eastern Finland Laboratory Center ISLAB. Insulin, apolipoprotein (Apo) A1, and ApoB concentrations were analyzed at the University of Eastern Finland.

### Responder and nonresponder definition

Participants who had a decrease in IHL content during the 12-week intervention were defined as responders (*N* = 13). Non-responders had unchanged or increased IHL during the intervention (*N* = 7).

### Stool samples

Stool samples were collected by the subjects themselves in a plastic container with a lid at the beginning and end of the study. The sealed container was brought to the research unit the next day in an ice box with ice bags. At the research unit, stool samples were directly homogenized, aliquoted, and frozen at −80°C without any detergents for further analysis. The frozen stool samples were sent to Novogene, United Kingdom, for DNA extraction and shotgun sequencing.

### DNA extraction and sequencing

The DNA extraction procedures were followed by QIAamp DNA Mini and Blood Mini Handbook (29). The DNA fragments were end-polished, A-tailed, and ligated with the full-length adapters of Illumina sequencing, followed by further PCR amplification with P5 and indexed P7 oligos. The PCR products as the final construction of the libraries were purified with the AMPure XP system. Then libraries were checked for size distribution by Agilent 2100 Bioanalyzer (Agilent Technologies, CA) and quantified by real-time PCR (to meet the criteria of 3 nM). The qualified libraries are fed into Illumina sequencers (NovaSeq system, 150-bp paired-end sequences).

### Metagenomics data processing

Trimmomatic was used to clip adapter and low-quality bases (v0.36, ILLUMINACLIP:TruSeq3-PE-2.fa:2:30:10:1:TRUE, LEADING:3, TRAILING:3, SLIDINGWINDOW:4:15, MINLEN:30). The remaining reads with less than 36-bp length were discarded. BWA (v07.17) was used to align quality-filtered reads to the human reference genome (hg38). Originally 20.1M ± 1.7M metagenomic reads remained after preprocessing per sample. MetaPhlAn3 and HUMAnN3 were used to estimate the taxonomic and Kyoto Encyclopedia of Genes and Genomes (KEGG) pathway composition of the non-human reads with default parameters. Low abundance species were removed at a cut-off = 0.05%. R packages vegan (v2.5) and picante (v1.8.2) were used to calculate alpha diversity.

### Differential correlation network analysis and enrichment analysis

We calculated the differentially correlated microbial pairs in the intervention and control groups, then excluded the differentially correlated microbial pairs that appeared in both groups. The remaining differentially correlated microbial pairs were used to construct the network of intervention groups. The abundance network was constructed based on the relative abundance values of all detected species (prevalence filter: 10%, abundance filter: 0.05%). DGCA (v2.0.0) was applied to construct the network from differentially correlated microbial pairs. To further decompose the complex microbiome network into sub-communities, MEGENA (v1.3.7) was used to identify modules in the constructed network using significantly different microbial pairs (*P* < 0.05). MEGENA performs clustering analysis through an iterative approach that divides parent clusters into sub-clusters and then evaluates the compactness and local clusteredness inside the cluster until no valid sub-clusters can be further identified. The same approach was also applied to analyze responder versus non-responder and to build the network of responder group.

We performed enrichment analysis, which includes permutation testing to determine whether correlations between modules and clinical parameters were possible by chance or not (30). The relative abundance of species was transformed by the centered log ratio transform method of the R package microbiome (v1.12.0). First, for a given module, correlations between particular clinical parameters and all species in this module were obtained using the partial Spearman correlation method adjusted by age, gender, and BMI. The sum of absolute correlation coefficients in this module was then calculated. Following that, the same number of species in the module was chosen at random 1,000 times from all species, and the sum of absolute values of every correlation was calculated for each set. Finally, the sum of significant correlation values in a given module was evaluated to determine whether it was higher than 95% of the sums of significant correlation values in the repeated random selected species.

### Statistics

#### Clinical

Unpaired *t*-test was used for comparing the clinical measurements between groups at baseline. For comparing both groups after 12 weeks, a generalized linear model (GLM) was used and adjusted by gender, age, BMI, and type 2 diabetes (T2D) status. Low-density lipoprotein cholesterol (LDL-C), gamma-glutamyl transferase (GGT), insulin, homeostatic model assessment-insulin resistance (HOMA-IR), and high-sensitive C-reactive protein (hs-CRP) were log10 transformed, and apolipoprotein (Apo) B and total cholesterol (TC) were square rooted for normalization.

#### Metagenomics

The R package vegan (v2.5) was used to calculate alpha diversity with Shannon and Chao index for each sample. Statistical differences in alpha diversity were obtained by the Wilcoxon rank-sum test (between groups) and Wilcoxon signed-rank test (within groups). For beta diversity, the R package phyloseq (v1.34.0) and coda.base (v0.3.1) were used to calculate the weighted and unweighted UniFrac distance and Aitchison distance for samples. Statistical difference of beta diversity was calculated by permutational multivariate analysis of variance (PERMANOVA). Metagenomseq (v1.32.0, zero-inflated Gaussian mixture model) was used to perform differential abundance analysis of species and pathways. Correlations between clinical parameters, species, and pathways were obtained using the partial Spearman correlation method adjusted by age, gender, and BMI. Distance-based redundancy analysis (dbRDA, from R package vegan) was used to analyze the relationship between the host clinical profile and the taxonomic and functional composition of the GM. For all statistical analyses, *P* < 0.05 was considered statistically significant. False discovery rate (FDR) was calculated to adjust *P* values for multiple hypotheses testing by applying Benjamini-Hochberg procedure.

## RESULTS AND DISCUSSION

### Exercise induced global and personalized changes in clinical and biochemical profiles of NAFLD patients

Forty-two subjects (25 women, 17 men) from Finland participated in this 12-week HIIT (details have been published before [28]). The subjects in the intervention (I) and control (C) groups were similar in age (I, 59.9 ± 9.8 years; C, 56.7 ± 10.7 years), BMI (I, 29.7 ± 3.2; C, 29.5 ± 4.3), and fitness levels measured as maximum oxygen consumption (VO_2_max) (I, 23.7 ± 4.0 mL/kg/min; C, 25.1 ± 5.3 mL/kg/min). During the intervention fasting, plasma glucose concentration and waist circumference decreased significantly (GLM, *P* < 0.05), whereas the maximum rate of oxygen consumption (VO_2_max) and maximum achieved workload (maxW) increased significantly compared to the control group (normalized to baseline, GLM, *P* < 0.05). The IHL content did not change significantly during the intervention in either group (GLM, *P* > 0.05). For the metagenomics analyses, stool samples from one subject in the intervention group and two subjects from the control group were excluded since these subjects used antibiotics during the intervention.

Interestingly, the IHL content in the intervention group increased in some participants, suggesting a heterogeneous response to exercise. Therefore, the intervention group (*N* = 20) was further sub-divided into responders (*N* = 13) and non-responders (*N* = 7) based on decreased or increased IHL content during the 12-week HIIT intervention. Baseline characteristics did not differ between the responders and nonresponders except for food intake (Table 1). A significantly higher intake of total fat and saturated fat (SFA) (*t*-test, *P* < 0.05) was reported at baseline in the nonresponder group compared to the responders.

**TABLE 1:** Clinical characteristics of responders and non-responders at baseline and at the end of the intervention^*d*^

Clinical parameter	Responder (baseline)	Non-responder (baseline)	Responder (12 wk)	Non-responder (12 wk)	*P* value^*a*^	*P* value^*b*^
Sex (M/F)	5/8	2/5				
Age, years	60.6 ± 10.6	59.4 ± 9.7			0.316	
IHL, %	17.61 ± 8.14	13.67 ± 10.82	14.1 ± 8.2	16.3 ± 11.6	0.394	**0**
Weight, kg	83.6 ± 19.2	85.5 ± 5.8	82.7 ± 19.6	86.3 ± 5.6	0.659	**0.009**
BMI, kg/m^2^	29.8 ± 3.7	29.8 ± 1.4	29.5 ± 3.8	30.1 ± 1.3	0.879	**0.008**
ALT, U/L	50.92 ± 21.2	48.14 ± 24.5	45.6 ± 23.1	48.3 ± 21.0	0.794	0.449
AST, U/L	34.23 ± 10.81	30.71 ± 7.8	34.2 ± 10.8	30.71 ± 7.8	0.458	0.811
GGT, U/L	66.4 ± 67.7	98.6 ± 76.1	69.5 ± 104.5	110.6 ± 86.0	0.344	0.081
TG, mmol/L	1.7 ± 0.8	1.7 ± 0.8	1.52 ± 0.65	1.96 ± 0.9	0.974	**0.015**
TC, mmol/L	4.4 ± 1.1	5.4 ± 1.0	4.0 ± 0.8	5.5 ± 1.2	0.06	0.081
LDL-C, mmol/L	2.7 ± 1.2	3.6 ± 1.1	2.3 ± 0.88	3.5 ± 1.09	0.108	0.076
HDL-C, mmol/L	1.4 ± 0.4	1.4 ± 0.4	1.44 ± 0.38	1.49 ± 0.5	0.964	0.394
Apo A1, g/L	1.54 ± 0.2	1.5 ± 0.2	1.53 ± 0.19	1.57 ± 0.29	0.902	0.289
Apo B, g/L	0.9 ± 0.32	1.1 ± 0.16	0.78 ± 0.28	1.15 ± 0.19	0.074	**0.028**
VFA, cm^2^	140.8 ± 45.5	163.5 ± 33.5	137.5 ± 42.1	170.4 ± 34.9	0.262	**0.03**
Fat mass, kg	29.3 ± 9.0	32.3 ± 6.4	28.9 ± 8.5	33.6 ± 4.6	0.432	**0.016**
Fat mass, %	34.7 ± 4.0	32.2 ± 4.8	34.7 ± 3.4	39.1 ± 6.0	0.184	**0.045**
Muscle mass, kg	30.3 ± 7.0	29.6 ± 4.9	29.9 ± 7.1	29.3 ± 4.8	0.822	0.959
Waist circumference, cm	100.4 ± 13.4	102.7 ± 7.0	99.7 ± 14.7	102.1 ± 6	0.681	0.846
Systolic BP, mm Hg	137.3 ± 9.0	141.1 ± 17.1	137.5 ± 7.6	136.9 ± 16.1	0.596	0.453
Diastolic BP, mm Hg	87.5 ± 6.0	91.6 ± 7.0	87.2 ± 4.7	88.1 ± 8.8	0.184	0.153
Gluc, mmol/L	6.4 ± 0.8	6.5 ± 1.1	6.3 ± 0.6	6.5 ± 0.9	0.765	0.89
HbA1c, mmol/L	40.3 ± 4.3	40.7 ± 7.6	40.2 ± 3.0	41.3 ± 8.2	0.879	0.487
Insulin, mU/L	18.8 ± 14.6	15.9 ± 12.6	17.2 ± 8.1	16.4 ± 10.0	0.654	0.596
HOMA-IR	5.3 ± 4.1	4.6 ± 3.7	4.7 ± 2.1	4.8 ± 3.2	0.707	0.600
hs-CRP, mg/L	1.2 ± 0.9	2.01 ± 0.01	2.6 ± 3.9	2.3 ± 0.9	0.09	0.878
VO_2_max, mL/minute	1.99 ± 0.6	1.97 ± 0.49	2.2 ± 0.7	2.1 ± 0.55	0.927	0.07
VO_2_max, mL/kg/minute	23.9 ± 3.8	22.9 ± 4.6	26.4 ± 4.1	24.0 ± 5.1	0.584	0.088
maxM, W	148.54 ± 53	152.86 ± 48.74	169.2 ± 51.3	166.5 ± 52.3	0.861	0.2
RMR, kcal/day	1,584 ± 322	1,440 ± 203	1,514 ± 190	1,591 ± 339	0.3	0.975
Energy, kJ	8,379 ± 1646	8,145 ± 2172	7,065 ± 1501	8,898 ± 2513	0.788	0.065
Energy, kcal	2,002 ± 393	1,946 ± 519	1,904 ± 358	2,125 ± 600	0.778	0.065
CHO, %^*c*^	41.7 ± 4.8	37.8 ± 5.7	42.0 ± 5.8	41.5 ± 5.7	0.123	0.236
Protein, %^*c*^	18.7 ± 4.0	17.9 ± 1.6	18.2 ± 4.2	15.4 ± 2.5	0.618	0.645
Fat, %^*c*^	34.8 ± 6.1	41.4 ± 5.9	36.7 ± 5.6	36.0 ± 3.9	**0.032**	**0.005**
SFA, %^*c*^	12.8 ± 2.6	15.8 ± 2.5	12.7 ± 3.1	14.0 ± 3.0	**0.021**	0.645
MUFA, %^*c*^	12.7 ± 2.5	14.9 ± 2.7	13.6 ± 2.3	12.0 ± 1.8	0.081	**0.012**
PUFA, %^*c*^	6.2 ± 1.3	6.2 ± 1.2	6.9 ± 1.5	5.6 ± 1.0	0.913	0.105
Cholesterol, mg	277 ± 87	310 ± 110	240 ± 49	310 ± 116	0.46	0.364
Fiber, g	24.6 ± 7.8	22.9 ± 6.9	24.0 ± 8.2	26.0 ± 6.5	0.6332	0.12

^*a*^ Comparing responder at baseline vs non-responder at baseline.

^*b*^ Comparing the changes of both groups after intervention; bold refers to significant.

^*c*^ From total energy intake.

^*d*^ M, male; F, female; T2D, type 2 diabetes; BMI, body mass index; ALT, alanine transferase; AST, asparagine transferase; GGT, gamma-glutamyl transferase; TG, triglyceride; TC, total cholesterol; LDL-C, low-density lipoprotein cholesterol; HDL-C, high-density lipoprotein cholesterol; VFA, visceral fat area; cf, circumference; BP, blood pressure; Gluc, glucose; HbA1c, glycated hemoglobin; HOMA-IR, homeostatic model assessment-insulin resistance; hs-CRP, high-sensitive C-reactive protein; VO_2_max, maximum rate of oxygen consumption = “cardiorespiratory fitness”; maxW, maximum workload achieved; RMR, resting metabolic rate; CHO, carbohydrate; SF, saturated fatty acids; MUFA, monounsaturated fatty acids; PUFA, polyunsaturated fatty acids.

During the 12-week intervention, there were significant differences in the fold changes of the concentrations of plasma triglycerides (TGs) and ApoB, visceral fat area and body fat mass in kilogram and percentage (normalized to baseline, GLM, *P* < 0.05; Table 1) when comparing responders with non-responders. There was also a significant change in body weight in the responders (GLM, *P* < 0.05); however, 900 g of weight loss in 12 weeks is not considered clinically significant. We observed a statistically non-significant trend toward reduced concentrations of TC, GGT, and LDL-C in the responders compared to non-responders (GLM, *P* = 0.081, 0.081, and 0.076, respectively). These trends are in line with previous aerobic exercise studies in NAFLD subjects, which have reported decreases in IHL and decreases in triglyceride (TG), ApoB, LDL-C, and TC concentrations (31 – 34). Although our results did not reach statistical significance, these findings may suggest that aerobic exercise has a favorable effect on lipid metabolism in NAFLD patients. Despite the instructions not to change the diet, the intake of total fat (GLM, *P* < 0.05) and monounsaturated fat (MUFA) (GLM, *P* < 0.05) was significantly different between the groups at the end of the 12-week intervention (Table 1). No significant differences were observed in SFA intake (GLM, *P* = 0.645) between the two groups during the intervention.

### *Microbiome variance* during exercise is associated with markers of liver and glucose metabolism

**Fig 1:**
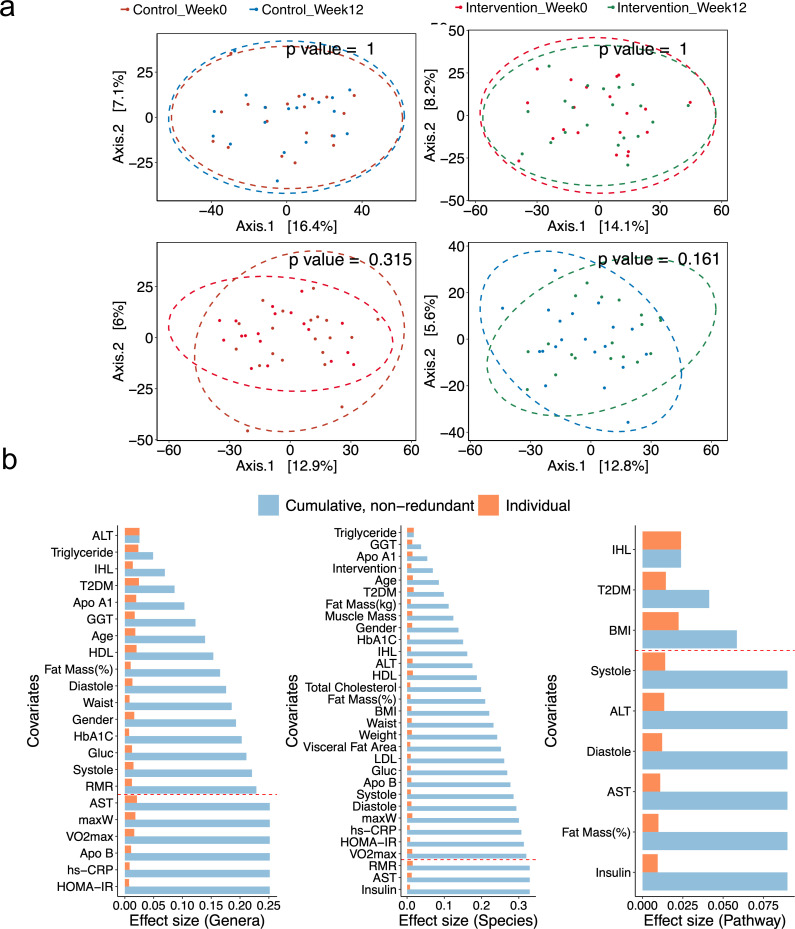
Global characteristics of gut microbiome and clinical profile. (a) Principal coordinate analysis (PCoA) of Aitchison dissimilarity between gut microbiome abundance profiles at the species level. PERMANOVA was used to assess the statistical significance of beta diversity comparisons within and between groups. (b) Clinical covariates explaining the variation of bacteria (left to right) genera, species, and pathways among all samples (*N* = 78, distance-based redundancy analysis). Aitchison distance was used to measure the beta diversity. The orange bars represent the individual variance explained by each of these covariates, while the cyan bars refer to the cumulative and non-redundant variance by stepwise dbRDA analysis. Only significant (*P* < 0.05) genera, species, and pathways in individual analysis are listed. Those above the red dotted line are signiﬁcant (*P* < 0.05) in the non-redundant analysis.

Shotgun metagenomics sequencing of stool samples from baseline and endpoint was used to examine the GM change during exercise. We sequenced an average of 20.1 gigabase pairs of high-quality reads per sample from 78 stool samples [control group at baseline (*N* = 20), control group at week 12 (*N* = 19), intervention group at baseline (*N* = 20), and intervention group at week 12 (*N* = 19)]. MetaPhlAn3 (35) was used for taxonomic profiling and identified 129 genera and 363 species across all samples. We measured the community alpha diversity as Shannon and Chao1 indices and calculated also the weighted/unweighted UniFrac distance and Aitchison distance of the control and intervention groups, at baseline and 12 weeks. However, there were no statistically significant differences induced by exercise within and between the groups (Wilcoxon signed-rank test, Wilcoxon rank-sum test, and PERMANOVA, *P* > 0.05) (Fig. 1a; Table S1).

Next, we applied dbRDA (from R package vegan) to evaluate the association of the metadata with the GM taxonomic and functional composition (Fig. 1b). Many significant correlations between the clinical variables and the GM variance were discovered in univariate analysis (dbRDA, *P* < 0.05). More than 20 covariates including the liver-related parameters, such as IHL, concentrations of aspartate aminotransaminase (AST), alanine transaminase (ALT), GGT, plasma total and lipoprotein lipids, ApoA1, ApoB, and fasting insulin and glucose, showed a significant correlation (dbRDA, *P* < 0.05) to the GM composition at the genus and species levels. Exercise parameters like VO_2_max and maxW, which were found to be significantly different (normalized to baseline, GLM, *P* < 0.05) between the intervention group and the control group, were found also significantly correlated with the species diversity. Moreover, IHL and concentrations of AST and ALT were significantly associated (dbRDA, *P* < 0.05) with the functional variance of the microbiota. In the non-redundant analysis, 16, 28, and 3 of the significant covariates accounted for 25.2%, 32.9%, and 9.0% non-redundant variance in the genus, species, and pathway profiles, respectively. Significant covariates like IHL, concentrations of ALT, GGT, TG, high-density lipoprotein cholesterol (HDL-C), fasting glucose, and VO_2_max and maxW had a significant correlation (dbRDA, *P* < 0.05) with the GM composition, and IHL remained significantly correlated (dbRDA, *P* < 0.05) with the functional variance of the microbiota.

**Fig 2:**
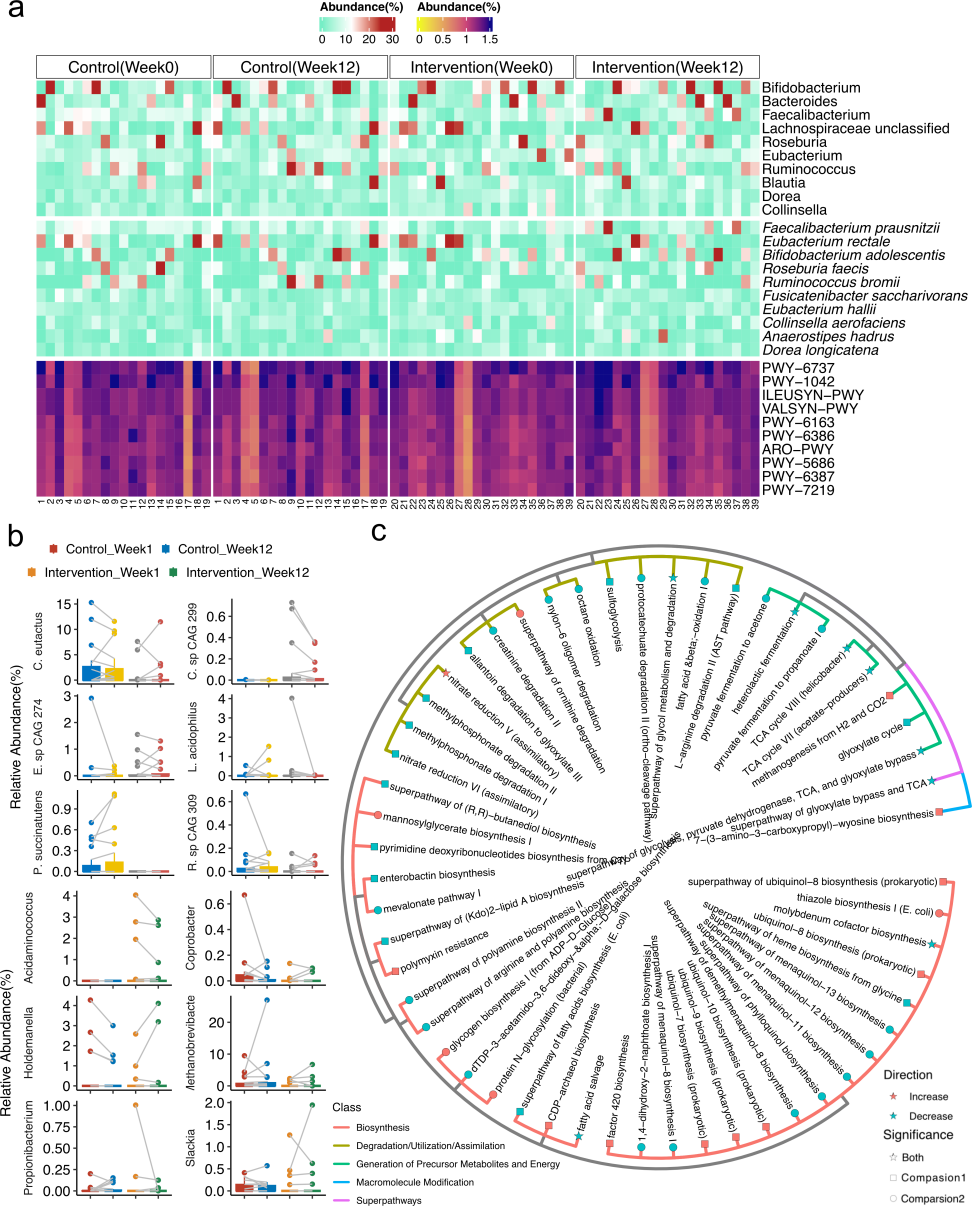
Top abundant genera/species/pathways and analysis of the differential abundance of genera/species/pathways in the intervention group. (a) Heatmap showing the relative abundance of the top 10 most abundant genera, species, and pathways in intervention and control group at baseline and week 12. (b) Selected significant genera/species with different abundance (zero-inflated Gaussian mixture model, *P* < 0.05). (c) Significant pathways with different abundance (zero-inflated Gaussian mixture model, *P* < 0.05). Two comparison ways were used for analyzing the differential abundance of genera, species, and pathways: Comparsion1, significant differential abundant species found between baseline and week 12 in the intervention group (but not significant in the control group); Comparsion2, significant differential abundant species found between intervention and control at week 12 (but not at baseline).

*Bacteroides*, *Dorea*, and *Ruminococcus*, known as short-chain fatty acid (SCFA) producers, which were found previously in higher abundances in NAFLD subjects compared to controls, were found as part of the 10 most abundant genera in our study (Fig. 2a) (6, 36, 37). *Faecalibacterium prausnitzii* found previously lower abundance in T2DM and obese people was the most abundant species in our subjects (Fig. 2a) (6). To find out the potential taxa responding to the exercise intervention, we analyzed (i) taxa whose abundance changed significantly only in the intervention group but not in the control group (zero-inflated Gaussian mixture model, *P* < 0.05) and (ii) taxa that differed significantly between the two groups at the end of the study but not at baseline (zero-inflated Gaussian mixture model, *P* < 0.05). We found in total seven genera and 19 species meeting these criteria; however, none of these 10 most abundant genera and species including *Bacteroides*, *Dorea*, *Ruminococcus* and *Faecalibacterium prausnitzii* was in this list (Fig. 2b; Table S2).

In addition, we identified 456 pathways across all samples using HUMAnN3 as the functional pathway’s profile annotation tool. Similar to the taxonomic profile, the functional potential of the microbiota was unable to distinguish within or between the groups based on alpha and beta diversity (PERMANOVA, *P* > 0.05) (Fig. 1a). We compared the individual pathways between the two groups using similar criteria as in the taxonomy profile. In total, 30 pathways met these criteria, yet none of the most abundant pathways including pathways previously associated with NAFLD, such as isoleucine and valine (PWY-6386, PWY-6387, ILEUSYN-PWY, and VALLSYN-PWY) (38), was part of this list of significantly different pathways (Fig. 2c).

### Exercise intervention altered significantly the gut microbiome interactome

To investigate the direct and indirect interactions within the bacterial community, we applied the R package DGCA (v2.0.0) (39), which takes the correlations between species in both before and after the exercise intervention into account and constructed a differential correlation network using the detected species in all samples. Our network displayed only the significant differential correlations among species (*P* < 0.05) and contained 66 nodes and 68 edges, representing the differentially correlated species pairs and the type of change in the observed correlations between these species, respectively (Fig. 2a see Materials and Methods). The exercise intervention induced 18 positive associations between species, including one association that was found negative before the exercise but turned positive during the 12-week intervention (−/+) and 17 that were not observed at baseline but only at week 12 (0/+). There were also 13 negative associations caused by the exercise intervention, including one that was positive at baseline but turned negative at week 12 (+/−) and 12 that were not seen at baseline but only at the week 12 (0/−). Additionally, 16 negative (−/0) and 21 positive (+/0) associations which were found at baseline were lost during the intervention. Overall, the direction and/or strength of the 68 correlation pairs implied that the exercise reshaped the bacterial interactions within the GM.

**Fig 3:**
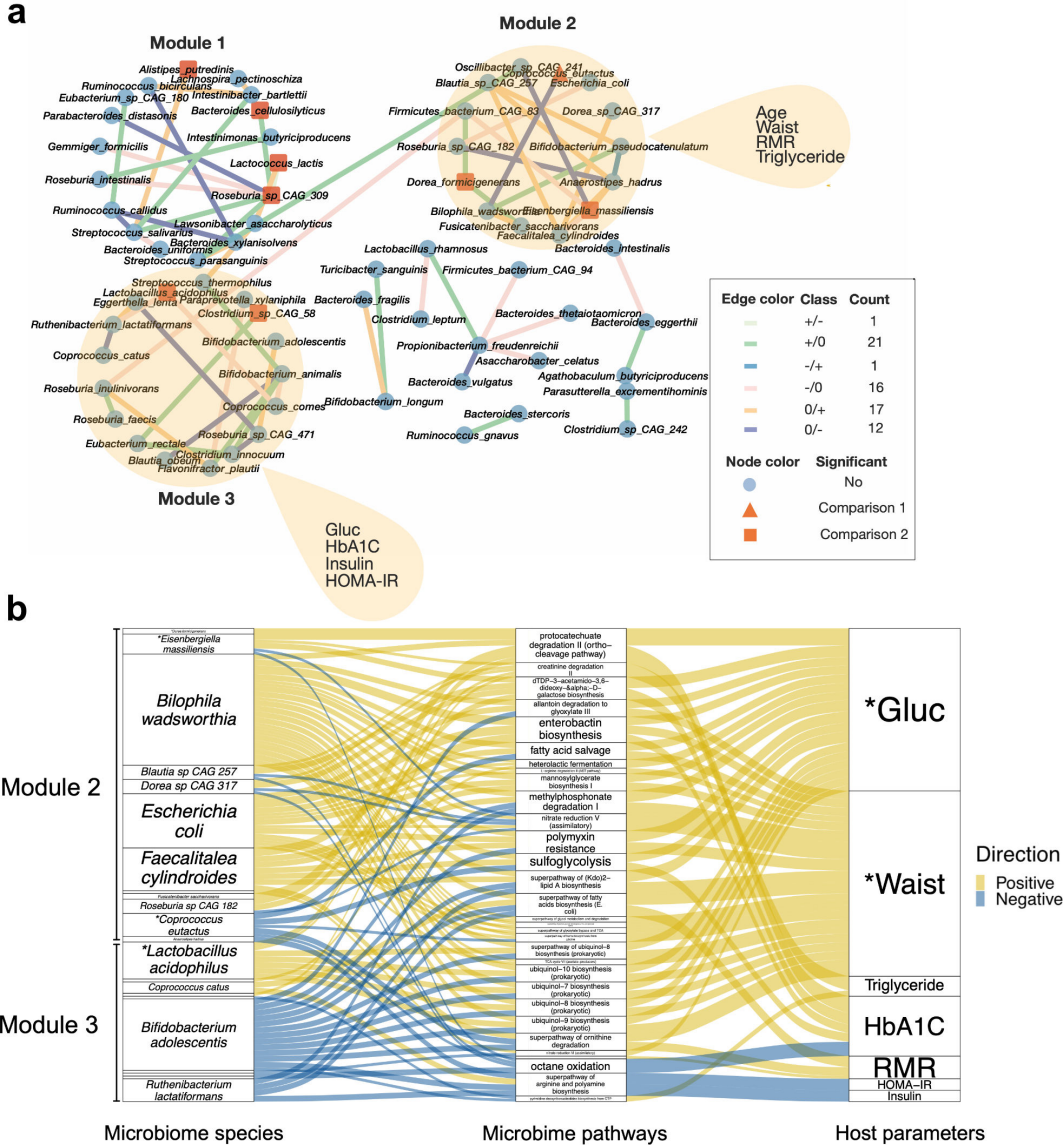
Analysis of microbiome-clinical associations in the intervention group. (a) Differential correlation network of the intervention group (see details in Materials and Methods). Triangle nodes refer to significant differential abundant species found between baseline and week 12 in the intervention group (but not in the control group), and square nodes refer to significant differential abundant species found between intervention and control at week 12 (but not at baseline). Significant differential species are colored in orange. (b) Alluvial plot was created using only samples in the intervention group, showing associations between bacterial modules, pathways, and clinical parameters.

Our network analysis revealed three GM species modules (Fig. 3a), from now on referred to as Module 1, Module 2, and Module 3. There were 18 species in Module 1, 14 species in Module 2, and 17 species in Module 3. We then surveyed the three modules for differentially abundant species (zero-inflated Gaussian mixture model, *P* < 0.05). We found the species *Alistipes putredinis*, *Bacteroides cellulosilyticus*, *Lactococcus lacti*, and *Roseburia* sp *CAG 309* in Module 1 to be significantly higher in the exercise intervention group compared to the control group (FDR = 0.02–0.14). *A. putredinis* and *L. lacti* were found decreased in a cohort study in cirrhosis patients and found to improve NAFLD progression in mice models (40, 41). In Module 2, we found *Coprococcus eutactus* significantly increased (Week 0 vs Week 12) in the exercise intervention group but not in the control group (FDR = 0.18). *C. eutactus* has been reported to be more abundant in healthy individuals than in individuals with NAFLD and suggested as probiotic bacteria against NAFLD (42). In the same module, we found *Dorea formicigenerans* and *Eisenbergiella massiliensis* having significantly higher relative abundance in the exercise intervention group when compared to the control (FDR = 0.044–0.145). Two species in Module 3 also showed significantly different abundances in the two groups: *Lactobacillus acidophilus* was found significantly lower in the exercise intervention group compared to the control group (FDR = 0.0008) and *Clostridium* sp *CAG 58* had significantly higher abundance in the intervention group compared to the control group (FDR = 0.14).

Next, we performed enrichment analysis in these three modules to evaluate the implication of GM restructuring following the exercise (see Materials and Methods section). No significant association was found between Module 1 and any clinical parameters. However, we found Module 2 had associations with waist, resting metabolic rate (RMR), and TG, and Module 3 had associations with concentrations of fasting glucose and insulin, HbA1c, and HOMA-IR (Fig. 3a). Therefore, we then specifically focused on Module 2 and Module 3 to study the association between their bacterial members, the significant GM functional pathways (zero-inflated Gaussian mixture model, *P* < 0.05), and the significant clinical parameters (GLM, *P* < 0.05) in more detail.

An alluvial plot (Fig. 3b) was used to integrate the connections between the bacterial modules, pathways, and clinical parameters. We observed a high number (81%) of positive correlations between the functional potential of the microbiome and Module 2. *Bilophila wadsworthia* and *Escherichia coli* in Module 2 had the most correlations. Many of the pathways were positively correlated with significant decreased plasma glucose concentration and waist circumference. A previous study showed that *B. wadsworthia* was positively correlated with fasting glucose concentration and aggravates high fat–induced metabolic functions including increased IHL content (43). In addition, there was a negative correlation between *B. wadsworthia* and *C. eutactus* observed after the exercise intervention (Fig. 3a), suggesting that decreased *B. wadsworthia* levels might facilitate the significant growth of *C. eutactus*. *C. eutactus* was previously found at lower levels in NAFLD subjects compared to healthy subjects and an increase could alleviate NAFLD progression (42, 44). The biosynthesis pathways of thiazole and polyamine and the nitrate reduction and protocatechuate degradation pathways had the highest number of correlations with the species in Module 2 (Fig. S2a, significantly correlated with at least five species, partial Spearman correlation, *P* < 0.05). It has been suggested that polyamine, nitrate, and protocatechuate play a role in obesity, NAFLD, and NASH (45 – 47).

Unlike Module 2, the majority of the correlations (66%) we observed between Module 3 and the microbiota pathways were negative. *Bifidobacterium adolescentis* and *Lactobacillus acidophilus* had the most correlations with the pathways. The relative abundance of *B. adolescentis*, a species that was reported to alleviate liver steatosis in mice (48), tended to increase during the 12-week intervention in the intervention group (*P* > 0.05). Interestingly, *L. acidophilus* which is known to be beneficial against NAFLD (49) decreased after the intervention in the intervention group (*P* < 0.05, FDR = 0.0008). The butanediol, menaquinol, methane, factor 420 biosynthesis pathways showed the most correlations with the species in Module 3 (significantly correlated with at least four species, partial Spearman correlation, *P* < 0.05) (Fig. S2a). Higher bacterial menaquinol production is associated with fibrosis in NAFLD patients (50). Furthermore, we found the protocatechuate degradation, which was positively correlated with *L. acidophilus* (partial Spearman correlation, *P* < 0.05), and the biosynthesis pathways of galactose, arginine, and polyamine mostly correlated with the clinical profile (significantly correlated with at least seven clinical parameters, partial Spearman correlation, *P* < 0.05) (Fig. S2b). Arginine was proposed to be a potential therapy for NAFLD when it conjugates with bile acids (51).

According to our differential correlation analysis, exercise had a significant impact on the microbial interactome. We identified three bacterial modules with a high number of differential correlations induced by exercise, which involved some significantly differential abundant species with a reported role in NAFLD progression. Modules 2 and 3 were correlated with clinical metabolic measurements associated with NAFLD, such as TG, insulin, and HOMA-IR (Fig. 2a), suggesting the potential implication of these bacterial consortia to synergistically affect clinical parameters during exercise.

### Network analysis suggested potential gut microbiota contributions in exercise responsiveness

We subsequently investigated the gut microbiota of the responders (13 subjects had decreased IHL) and non-responders (seven subjects had increased IHL) within the exercise intervention group using differential correlation network and enrichment analysis as above. The species’ alpha and beta diversity showed no significant difference within or between the responders and non-responders (Fig. S3). The responder network displaying only the significant differential correlations (*P* < 0.05) contained 43 nodes and 34 edges. The exercise intervention resulted in total of 16 new correlations in the responders, including three associations that were not observed at baseline but became positive at week 12 (0/+) and 13 that were not observed at baseline but found negative at week 12 (0/−). Also, 18 correlations were lost during the intervention, including nine that were found negative at baseline but were lost during the 12-week intervention (−/0) and nine that were positive at baseline but disappeared during the 12-week intervention (+/0).

**Fig 4:**
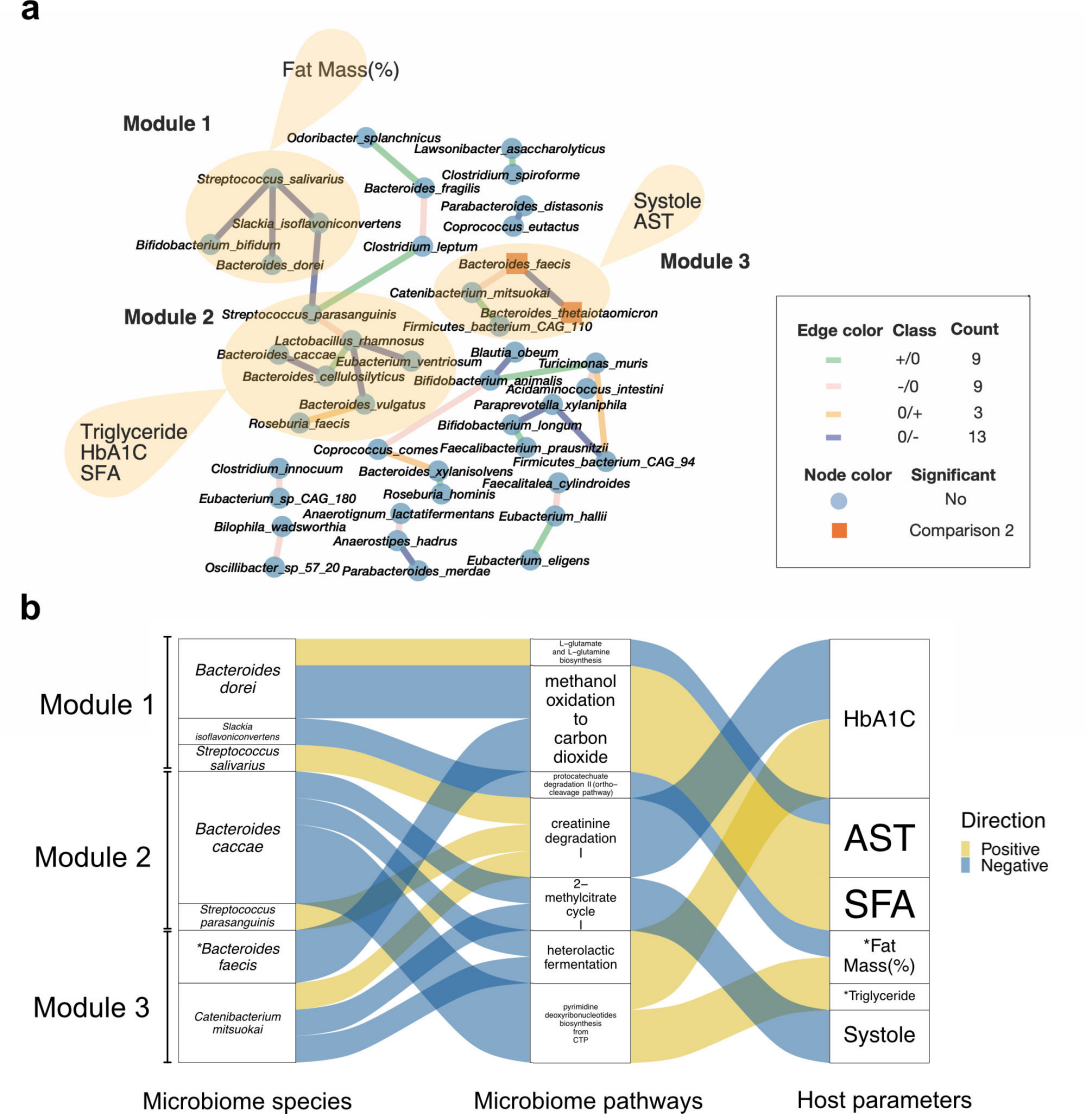
Analysis of microbiome-clinical associations in the responder group. (a) Differential correlation network of the responder group (see Materials and Methods). Square nodes refer to significant differential abundant species found between responders and non-responders at week 12 (but not at baseline). Significant differential species are colored in orange. (b) Alluvial plot was created using only samples in the responder group, showing associations between bacterial modules, pathways and clinical parameters.

This differential correlation network also revealed three GM modules in the responder group (Fig. 4a). There were four species in Module 1, seven species in Module 2, and four species in Module 3. The enrichment analysis in the three modules revealed that Module 1 was significantly associated with fat mass percentage; Module 2 had significant associations with plasma TG concentration, HbA1c, and intake of SFA; and Module 3 was significantly associated with systolic blood pressure and plasma asparagine transferase (AST) concentration. We examined the species abundances present in these modules using similar criteria as in the analysis above for the exercise intervention group. We found no species in Module 1 and Module 2 with statistically significant abundance changes. In Module 3, we found two species significantly different in relative abundance between the responder and non-responder group at the end of the study but not at baseline, including *Bacteroides thetaiotaomicron* and *Bacteroides faecis*, which were significantly lower and higher in responder, respectively (zero-inflated Gaussian mixture model, log2FC = −0.23 and 1.46, *P* < 0.05, FDR = 0.056 and 0.091; Table S3).

We subsequently built an alluvial plot (Fig. 4b) integrating the bacterial modules, functions, and clinical parameters. The strongest influence on clinical parameters in Module 1 came from *Bacteroides dorei*. This bacterium is shown to lower lipopolysaccharide production in the gut (52). The pathways of creatinine and protocatechuate degradation had significant correlations with the species in Module 1 (partial Spearman correlation, *P* < 0.05; Fig. S4a). There were previous studies suggesting that creatinine and protocatechuate are associated with NAFLD (47, 53). HbA1c, concentrations of fasting glucose, ALT and GGT, visceral fat area, and fat mass were also significantly negatively correlated with these pathways (partial Spearman correlation, *P* < 0.05; Fig. S4b).

The main contributors of Module two impacting the clinical parameters included *B. caccae* and *Bacteroides cellulosilyticus*. Both were slightly decreased in the responder group. These two species were found to be higher in advanced fibrosis in NAFLD (54, 55), but *B. cellulosilyticus* seems also to be associated with a healthy fasting lipid profile (56). The increased alanine fermentation (to propionate and acetate) pathway, which is negatively associated with concentrations of AST, insulin, LDL-C and GGT, weight, fat mass, and visceral fat area, was correlated with bacteria only from Module 2 (Fig. 4b; Fig. S4b).

*B. faecis* and *Catenibacterium mitsuokai* in Module 3 had the most associations with the pathways. We found *B. faecis* significantly increased at week 12 in the responder group (zero-inflated Gaussian mixture model, *P* < 0.05) and was also negatively correlated with the methanol degradation pathway, which has associations with decreased concentrations of liver enzymes ALT and AST (Fig. S4b). *B. faecis* was also found previously to be increased after decreased liver fat and was negatively associated with inflammatory markers such as monocyte chemoattractant protein-1 (MCP-1), chemokine ligands 4 (CCL4), matrix metalloproteinase-1 (MMP-1), and tumor necrosis factor-alpha (57). Moreover, a negative correlation between insulin resistance and this bacterium was noticed in another study (56). There was a negative association between *B. faecis* and *B. thetaiotaomicron* (Fig. 4a). *B. thetaiotaomicron* from Module 3, which significantly decreased in responders, was significantly correlated with glycogen degradation (partial Spearman correlation, *P* < 0.05).

We took a closer look into the functional profile and specifically the associations between species and pathways, and the associations between pathways and clinical parameters (Fig. S4). We observed that creatinine degradation pathway, which was negatively correlated with HbA1c and fasting glucose concentration, showed the most correlations with the species in the three modules (significantly correlated with at least three species, partial Spearman correlation, *P* < 0.05). The gut microbiota functional potential in methanol oxidation and alanine fermentation (to propionate and acetate) had the most correlations with the clinical profile (significantly correlated with at least seven clinical parameters, partial Spearman correlation, *P* < 0.05). *B. dorei* and *B. faecis* were significantly negatively correlated with the methanol oxidation pathway.

Previous microbiota network analyses in diseases showed the importance of the microbial interactome. For example, disturbances in the microbial network of Crohn’s disease might affect the relapse of the disease and non-response to antibody treatment (19). In addition, a recently published study about the microbial interactome in subjects with bronchiectasis exacerbations revealed differences in the numbers and directions of interactions of shared microbes between low- and high-frequency exacerbation clusters (9). This suggests that not the abundance of the microbe but more the interaction of the microbe with others are relevant for the disease outcome (9).

Our study uncovered besides differences in abundances of microbes in the intervention and control, changes in the bacterial interaction pattern. Species such as *B. wadsworthia* that are not significantly different between the groups but has several interactions with the pathway influenced the significant clinical outcomes (Fig. 4) and has also interaction with a significantly changed species (*C. eutactus*) suggesting an important player in the improvement of NAFLD progression performing exercise besides its absence of significance abundance between and within the groups. In addition, species in the interactome of responders and non-responders (e.g., *Catenibacterium mitsuokai*) were also non-significant in the richness but interacted with a significantly changed species (*B. faecis*), and both were associated with pathways that correlated with clinical outcomes in this module (Fig. 4). Therefore, the microbiota richness alone might not be a sufficient indicator of microbes’ impact on the microbiome and clinical events (9) but rather cooperation and competition in their ecosystem. Finally, the small cohort size of our study may have prevented us from detecting some species with significant interactions with clinical parameters in the abundance analysis. Despite this limitation, our study revealed significant changes in the bacterial interaction pattern, highlighting the potential importance of microbial cooperation and competition in the microbiome and clinical outcomes.

### Conclusion

We retrieved stool samples from a 12-week controlled HIIT intervention in NAFLD subjects (28) to investigate the changes in the gut microbiota induced by exercise as well as the role of the gut microbiota as a mediator of the positive effects of exercise in clinical parameters reflecting liver damage. While most microbiome research has focused on finding individual species that may be involved in the development of human diseases, believe that complex diseases, including NAFLD, would be more successfully treated with consortia of species especially considering that bacterial functionality is not only dependent on its own genomic information but also influenced by the interaction with other microbes (58). From our differential correlation network analyses, we found that the microbial interactome was significantly altered by exercise and revealed small modules of bacteria potentially involved in the host’s metabolic responsiveness to exercise. Even though our study was a carefully conducted randomized controlled exercise intervention and all HIIT sessions were supervised, it has limitations. The sample size was relatively small. In addition, whereas the GM interactome was thoroughly studied, conclusions for the newly formed interactomes cannot be easily drawn, since the co-occurrence analysis is undirected and unweighted (59). More research is needed to understand the causative links between non-responder microbiome and responsiveness to exercise in NAFLD patients to design combinatorial therapeutic approaches.

## Data Availability

Strengthening The Organization and Reporting of Microbiome Studies (STORMS) checklist (60) and raw metagenomic sequencing data for all samples have been deposited in European Nucleotide Archive under accession ID PRJEB56669. Further information and requests for resources and reagents should be directed to G. Panagiotou.

## SUPPLEMENTAL MATERIAL

Supplemental figures and tables msystems.00224-23-s0001.docx Fig. S1-S4 and Tables S1-S3.

ASM does not own the copyrights to Supplemental Material that may be linked to, or accessed through, an article. The authors have granted ASM a non-exclusive, world-wide license to publish the Supplemental Material files. Please contact the corresponding author directly for reuse.
