# Supplementary material for: Gut ecological networks reveal associations between bacteria, exercise, and clinical profile in non-alcoholic fatty liver disease patients
Source: mSystems. 2023 Aug 22;8(5):e00224-23. doi: 10.1128/msystems.00224-23 (PMC10654067; doi:10.1128/msystems.00224-23)
Supplement: Supplemental figures and tables — Fig. S1-S4 and Tables S1-S3. [file msystems.00224-23-s0001.docx]

Supplementary

**Table S1. Comparison of the gut microbiome profiles between intervention group and control group.** Weighted and unweighted UniFrac dissimilarity between gut microbiome abundance profiles at species level. P values are shown.

| Comparison | Weighted UniFrac | Unweighted UniFrac |
| --- | --- | --- |
| Control_week0 vs Intervention_week0 | 0.708 | 0.224 |
| Control_week12 vs Intervention_week12 | 0.881 | 0.192 |
| Intervention_week0 vs Intervention_week12 | 1 | 1 |
| Control_week0 vs Contral_week12 | 0.999 | 1 |

**Table S2. Significant differential genera and species in intervention versus control.** Two comparisons including (i) significant differentially abundant taxa (P < 0.05) found between baseline and week 12 in the intervention group (but not in the control group), (ii) and significant differentially abundant taxa (P < 0.05) found between intervention and control at week 12 (but not at baseline).

| **significant differentially abundant taxa found between baseline and week 12 in the intervention group (but not in the control group)** | | | |
| --- | --- | --- | --- |
|  | pvalues | adjPvalues | Log2FC |
| Lactobacillus_acidophilus | 0.000768164 | 0.093715996 | -6.116810294 |
| Coprococcus_eutactus | 0.003030411 | 0.184855052 | 0.413797524 |
| Roseburia_sp_CAG_309 | 0.009284052 | 0.377551438 | 0.538769831 |
| Bacteroides_intestinalis | 0.017588357 | 0.53644489 | 0.929137694 |
| Turicibacter_sanguinis | 0.026410216 | 0.644409274 | -1.360640324 |
| Ruminococcus_lactaris | 0.034530496 | 0.702120078 | 0.803638938 |
| Bacteroides_eggerthii | 0.041251273 | 0.718950757 | 1.122335026 |
| Holdemanella_biformis | 0.04829244 | 0.736459704 | 0.93150762 |
| **significant differentially abundant taxa found between intervention and control at week 12 (but not at baseline)** | | | |
|  | pvalues | adjPvalues | Log2FC |
| Lactobacillus_acidophilus | 2.48E-05 | 0.000821111 | -5.243583995 |
| Eubacterium_sp_CAG_274 | 2.95E-05 | 0.000821111 | 2.348213782 |
| Clostridium_sp_CAG_242 | 0.001718784 | 0.016526769 | -0.091319154 |
| Bacteroides_caccae | 0.002293554 | 0.020478161 | -1.466907937 |
| Roseburia_sp_CAG_309 | 0.003207002 | 0.026725016 | -0.838195795 |
| Adlercreutzia_equolifaciens | 0.005181037 | 0.038095864 | 1.369088172 |
| Asaccharobacter_celatus | 0.005876464 | 0.038660945 | 1.481821854 |
| Eubacterium_sp_CAG_251 | 0.007343649 | 0.044104033 | 1.191492175 |
| Dorea_formicigenerans | 0.007409478 | 0.044104033 | 0.838841784 |
| Bacteroides_cellulosilyticus | 0.007960843 | 0.045232064 | -0.216147918 |
| Alistipes_inops | 0.011940384 | 0.062189499 | 0.250399106 |
| Catenibacterium_mitsuokai | 0.01441671 | 0.07208355 | 0.615987022 |
| Lactococcus_lactis | 0.027029069 | 0.125134578 | -2.712296266 |
| Holdemanella_biformis | 0.029323564 | 0.126394674 | 0.022948917 |
| Alistipes_putredinis | 0.03605314 | 0.139146821 | -0.850003914 |
| Clostridium_sp_CAG_58 | 0.036734761 | 0.139146821 | 1.083454114 |
| Eisenbergiella_massiliensis | 0.039916011 | 0.144542818 | 2.898441084 |

**Table S3. Significant differential genera and species in responders versus non-responders.**

Two comparisons including (i) significant differentially abundant taxa (P < 0.05) found between baseline and week 12 in the responders (but not in the non-responders), (ii) and significant differentially abundant taxa (P < 0.05) found between responders and on-responders at week 12 (but not at baseline).

| **significant differentially abundant taxa found between baseline and week 12 in the responders (but not in the non-responders)** | | | |
| --- | --- | --- | --- |
|  | pvalues | adjPvalues | Log2FC |
| Eubacterium_sp_CAG_38 | 0.002016357 | 0.221799295 | -1.157463385 |
| Prevotella_copri | 0.025155415 | 0.864580036 | 3.961084568 |
| Blautia_sp_CAG_257 | 0.026887453 | 0.864580036 | 0.906188615 |
| Bacteroides_xylanisolvens | 0.041832882 | 0.864580036 | -1.089888725 |
| **significant differentially abundant taxa found between responders and non-responders at week 12 (but not at baseline)** | | | |
|  | pvalues | adjPvalues | Log2FC |
| Clostridium_disporicum | 6.10E-06 | 0.000229835 | -5.008754038 |
| Eubacterium_sp_CAG_38 | 5.42E-05 | 0.001156675 | -0.830680296 |
| Methanobrevibacter_smithii | 0.000226454 | 0.003198665 | -0.267663514 |
| Prevotella_copri | 0.000823023 | 0.008454686 | 1.844568916 |
| Bifidobacterium_animalis | 0.003477087 | 0.029281742 | -1.877755258 |
| Alistipes_inops | 0.003627826 | 0.029281742 | 1.736380417 |
| Bacteroides_stercoris | 0.004682982 | 0.03307356 | -0.827155688 |
| Ruminococcus_lactaris | 0.005414144 | 0.035463205 | 1.940096957 |
| Bacteroides_faecis | 0.010474435 | 0.056362435 | -1.638878304 |
| Firmicutes_bacterium_CAG_145 | 0.011443712 | 0.058779066 | 2.241519394 |
| Holdemanella_biformis | 0.012716125 | 0.062453593 | -0.470591987 |
| Parasutterella_excrementihominis | 0.01326448 | 0.062453593 | 1.51502924 |
| Faecalitalea_cylindroides | 0.015306945 | 0.069187391 | -4.80880112 |
| Bacteroides_thetaiotaomicron | 0.023337496 | 0.090935761 | 3.476654341 |
| Eubacterium_eligens | 0.037052039 | 0.135060657 | 1.328613197 |
| Phascolarctobacterium_sp_CAG_266 | 0.042562063 | 0.150297286 | Inf |
| Lachnospira_pectinoschiza | 0.044935121 | 0.153868749 | -0.929763744 |


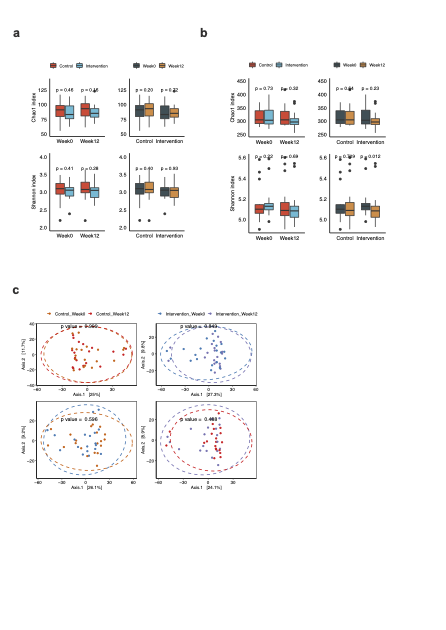


**Fig S1. Comparison of the gut microbiome profiles between intervention group and control group.**

**(a)** Alpha diversities (Shannon and Chao1) of gut microbiome species. Box plots showed median (center lines), lower/upper quartiles (box limits) and whiskers (the last data points 1.5 times interquartile range (IQR) from the lower or upper quartiles). **(b)** Similar alpha diversity box plots of the functional pathway profiles. **(c)** Principal coordinate analysis (PCoA) of Aitchison dissimilarity between functional pathway profile.

**Fig S2. Correlation heatmap of gut microbiome profiles and clinical parameters in intervention group.** Heatmaps were created using only samples in the intervention group. Significant pathways and species were obtained by comparing baseline and week 12 in the intervention group. The direction of species, pathways and clinical parameters in heatmaps represents whether there was an increase or decrease in the week 12 when compared to baseline. (**a**) Heatmap showing the correlations between significant pathways and species from bacterial modules which showed significant association with clinical parameters. (**b**) Heatmap showing the correlations between clinical parameters and significant pathways. Significant correlations in heatmaps were denoted as asterisks (“*”, P < 0.05).

**Fig S3. Comparison of the gut microbiome profiles between responder and non-responder.** **(a)** Alpha diversities (Shannon and Chao1) of gut microbiome species. Box plots showed median (center lines), lower/upper quartiles (box limits) and whiskers (the last data points 1.5 times IQR from the lower or upper quartiles). **(b)** PCoA of Aitchison dissimilarity between gut microbiome abundance profiles at species level.

**Fig S4. Correlation heatmap of gut microbiome profiles and clinical parameters in responder group.** Heatmaps were created using only samples in the responder group. Significant pathways and species were obtained by comparing baseline and week 12 in the intervention group. The direction of species, pathways and clinical parameters in heatmaps represents whether there was an increase or decrease in the week 12 when compared to baseline. (**a**) Heatmap showing the correlations between significant pathways and species from bacterial modules which showed significant association with clinical parameters. (**b**) Heatmap showing the correlations between clinical parameters and significant pathways. Significant correlations in heatmaps are denoted as asterisks (“*”, P < 0.05).
